# Recent major changes to TAIR: updates to the database, website, and *Arabidopsis* genome

**DOI:** 10.1093/genetics/iyaf248

**Published:** 2026-01-01

**Authors:** Leonore Reiser, Alyssa Proia, Erica Bakker, Shabari Subramaniam, Kartik Khosa, Swapnil Sawant, Xingguo Chen, Trilok Prithvi, Tanya Z Berardini

**Affiliations:** Phoenix Bioinformatics, 39899 Balentine Drive, Suite 200, Newark, CA 94560, United States; Phoenix Bioinformatics, 39899 Balentine Drive, Suite 200, Newark, CA 94560, United States; Phoenix Bioinformatics, 39899 Balentine Drive, Suite 200, Newark, CA 94560, United States; Phoenix Bioinformatics, 39899 Balentine Drive, Suite 200, Newark, CA 94560, United States; Phoenix Bioinformatics, 39899 Balentine Drive, Suite 200, Newark, CA 94560, United States; Phoenix Bioinformatics, 39899 Balentine Drive, Suite 200, Newark, CA 94560, United States; Phoenix Bioinformatics, 39899 Balentine Drive, Suite 200, Newark, CA 94560, United States; Phoenix Bioinformatics, 39899 Balentine Drive, Suite 200, Newark, CA 94560, United States; Phoenix Bioinformatics, 39899 Balentine Drive, Suite 200, Newark, CA 94560, United States

**Keywords:** knowledgebase, gene function, gene annotation, plant genomics, data integration, database, biocuration, *Arabidopsis thaliana*

## Abstract

The *Arabidopsis* Information Resource (TAIR) is the primary knowledgebase for *Arabidopsis thaliana*, providing curated genetics and genomics data, analysis tools, and community resources that support plant biology research worldwide. Since its establishment in 1999, TAIR has continuously evolved to meet the growing needs of the research community, with data curation drawn from the peer-reviewed literature, integration of community contributions, and development of search, visualization, and analysis tools. In this update, we highlight major advances since 2024, including the complete replatforming of TAIR to a modern, microservices-based architecture with a redesigned user interface, improved search and browsing capabilities, and streamlined bulk retrieval functions. These updates have enhanced scalability, performance, and compliance with emerging web accessibility standards. We also describe the community-driven reannotation of the *A. thaliana* Col-0 reference genome (TAIR12), which integrates new long-read assemblies, updated structural annotations, and expert manual review and involved contributions by over 100 scientists worldwide. This effort delivers the most complete and accurate reference genome to date, strengthening the foundation for functional and comparative genomics. Sustained by a decade-long user-backed funding model, TAIR continues to provide weekly database updates and quarterly data releases while adapting its subscription framework to ensure equitable access for individuals, institutions, and educators. Looking ahead, we outline plans for incorporating artificial intelligence into curation and user support, with possibilities including AI-assisted literature triage, annotation, and query navigation. Together, these developments reinforce TAIR's role as a cornerstone of plant biology and a model for sustainable, community-driven knowledgebases.

## Introduction

The *Arabidopsis* Information Resource (TAIR) is a knowledgebase for the model plant *Arabidopsis thaliana* that consolidates information about its genetics and genomics into a central web accessible resource (Reiser et al. 2024). Its curation staff extracts, organizes, and interconnects information from published, peer-reviewed literature to constantly expand the corpus available for discovery by the plant biology and broader scientific community. It provides resources for searching and browsing data at the single gene level, for groups of genes, and for the entire genome. TAIR also provides data visualization tools, such as genome browsers, and data analysis tools, such as BLAST.

TAIR has been a community resource since its inception—integrating community feedback, loading datasets, vetting submissions, managing outreach and education for users, and supporting teachers who integrate TAIR into their learning modules. Feedback is gathered in person at annual conferences such as the International Conference on *Arabidopsis* Research, the Plant Biology meeting organized by the American Society for Plant Biologists, and the Plant and Animal Genomes meeting, as well as through email, social media, and user surveys.

In this publication, we summarize advances made in the last 2 yrs and discuss upcoming developments. TAIR has coordinated the reannotation of the *A. thaliana* reference genome and migrated to a new infrastructure for the website, all while continuing to curate information from the primary literature. We assess TAIR's now decade-old sustainability model. Finally, we discuss our plans for the applications of artificial intelligence (AI), including Agentic AI, for the database.

## A brief overview of TAIR

**Fig. 1.**
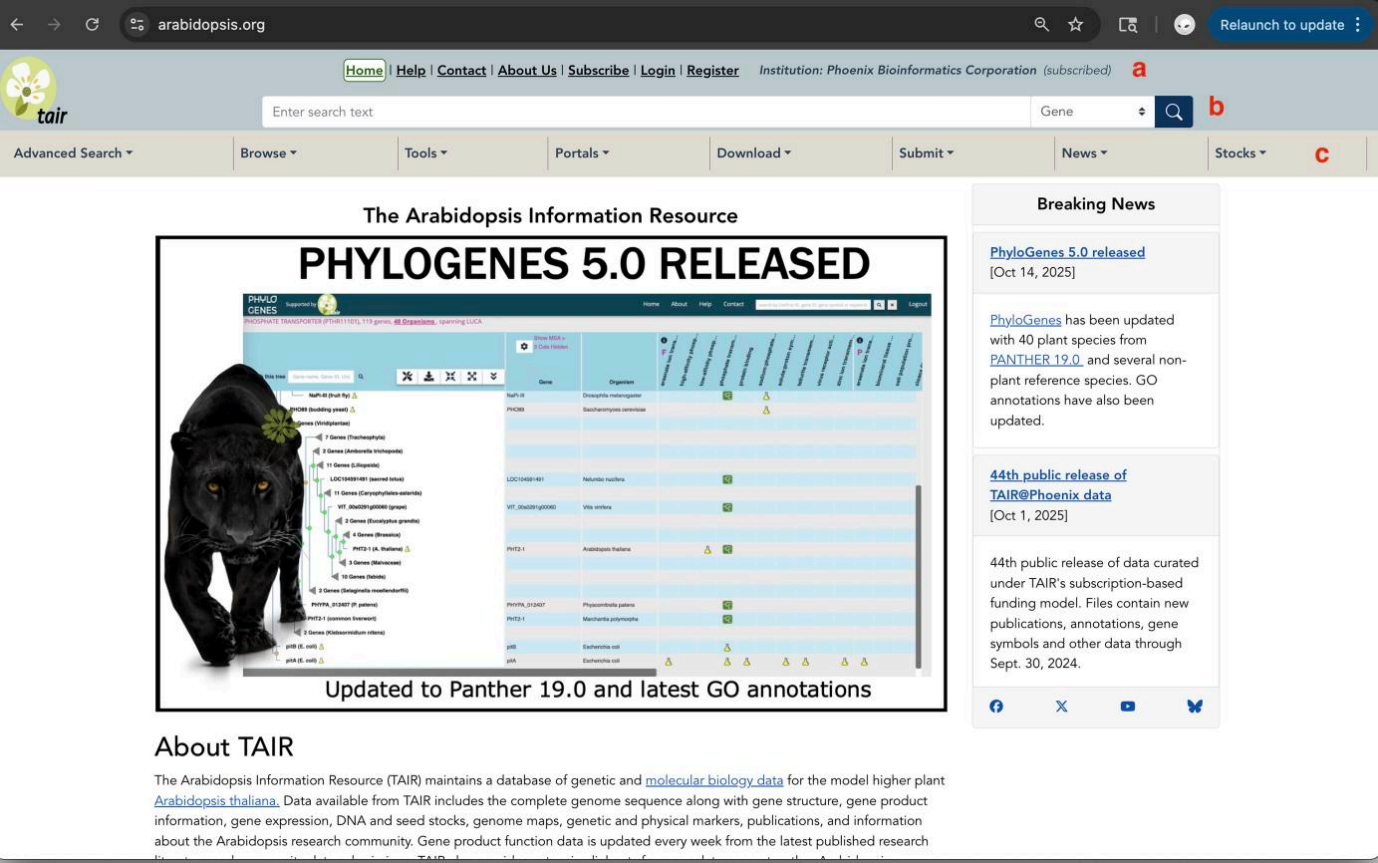
TAIR home page and navigation features (a) quick links to help resources, registration, and user profile management tools, b) general search in the toolbar, c) header drop-down menus for major content areas: Advanced Search, Browse, Tools, Portals, Download, Submit, News, and Stocks.

TAIR is a knowledgebase centered on curated information about the *Arabidopsis* reference genome and a community hub for researchers. Table 1 indicates the number and type of frequently accessed data types/datasets in TAIR. Curated experimental data is presented to users via the TAIR website, which is organized into major content areas (Fig. 1).

**Table 1.** Some data types and data sets in TAIR as of August 2025.

Data type	Number
Locus pages	40,569
Phenotypes	18,089
Papers linked to genes	53,025
Quarterly releases	43
Gene symbols linked to loci	30,369 symbols linked to 18,449 loci
Alleles and polymorphisms	1,965,337
Curated locus summaries	19,022
GO annotations (all)	225, 927
GO annotations (experimental)	99,252

### Search

TAIR curates information centered around various data objects from peer-reviewed scientific literature. These organized data can be queried through a General Search (Fig. 1b) in the site header as well as through advanced search interfaces (Fig. 1c) that allow narrowing the search space by various criteria.

### Browse

Various types of information are available for browsing. These include information that is regularly updated such as Gene Symbols that are registered with TAIR, Transposon Families, and Recently Added Literature, as well as legacy data sets like community-contributed Gene Families and Microarray Experiments.

### Tools

This section provides links to both in-house and external tools for *Arabidopsis* data visualization and analysis. TAIR provides several genome browsers for visualization of genes and other data that can be mapped to a chromosome assembly. JBrowse was introduced in May 2020, with JBrowse2 following in November 2023. TAIR will be ending support for the legacy tools GBrowse and SeqViewer after the update of the official genome annotation to TAIR12 (see the following section). Sequence analysis tools include BLAST, PatMatch (regular expression search for finding short sequences), and Motif Analysis. Other local visualization tools include PhyloGenes (Zhang et al. 2020), TAIR's in-house tool for visualizing phylogenetic trees along with functional annotation data for protein function prediction; Synteny Viewer for displaying precomputed syntenic relations between *Arabidopsis* and dozens of other plant species; and a Chromosome Map tool. Examples of links to external tools include GO Term Enrichment (sends requests to PANTHER [Mi et al. 2019; Thomas et al. 2022]; AraCyc (redirects to the Plant Metabolic Network [Hawkins et al. 2025]) for searching metabolic pathways databases; and Textpresso (Müller et al. 2018) for full-text searching a subset of the *Arabidopsis* literature corpus.

### Portals

TAIR is a community resource providing curated collections of information relevant to the *Arabidopsis* Research community. The portal pages aggregate information about resources like Gene Expression databases, analysis and visualization software, and Stock Centers to help researchers find reagents to aid in their research. New listings can be readily added, and we encourage the community to offer suggestions for resources to include.

### Downloads

This section provides access to all downloadable files, including new data releases and older, archived datasets. TAIR's curated functional annotation data is made available in quarterly releases. Subscribed users get access to the most recent releases (Subscriber Data Releases); all other users can access year-old data in the Public Data Releases folder. The public data releases are also mirrored in TAIR's Zenodo repository (Berardini et al. 2024). TAIR's Gene Ontology Annotations are made available to the public on a quarterly basis and are incorporated into the GO Consortium database. Current and archived genome releases can be found in the Genes directory.

### Data submission

As a first step for making published data Findable, Accessible, Interoperable and Reusable (FAIR), researchers need to know where and how to submit their data. The data submission section provides information to users about what data they can submit to TAIR and where they should submit other types of Arabidopsis data that TAIR does not directly accept.

### News, job postings, and events

TAIR also maintains public job listings, event postings, and news updates that are posted to the website and TAIR's social media accounts.

### Help and other documents

The Help and About sections of the website provide important information about TAIR, including how to use the database and tools, how to cite the resources, and how to link to it.

## Overhaul of the TAIR user interface and data retrieval infrastructure

### Why was an overhaul necessary?

On May 26, 2024, TAIR released a completely overhauled user interface (UI) for the website.

This modernization was both a challenge and an opportunity for our small but dedicated team at Phoenix Bioinformatics. The legacy system, built over two decades ago (Rhee et al. 2003; Weems et al. 2004), had accumulated technical limitations that impacted performance, speed, and the ability to scale. Though we had migrated all the TAIR resources (website, data server, tools) from physical servers under our direct management to AWS in November 2015, the underlying software was not updated. Beginning in January 2022, with the limited resources typical of a small nonprofit, our team embraced the updating challenges and set out to future-proof TAIR.

When TAIR was created in 1999, it was originally implemented using an object-oriented (OO) approach to data representation (Weems et al. 2004). The object-oriented design of the database integrated very naturally with Java, the object-oriented programming language, with the initial tools and data pages run as servlets, Java programs running on the server. The Apache web server forwarded HTTP requests to these servlets. Perl was used for many CGI programs at TAIR, such as the initial stages of the database queries and the BLAST and FASTA searches.

In 2011, the database was migrated to Oracle, and the middleware was refactored to take the new database into account. In 2015, the system migrated once more to Oracle hosted in the Amazon Web Services (AWS) Cloud. However, the basic system architecture continued to be monolithic. As staff transitions brought newer team members with different technical backgrounds, maintaining a resource built on legacy technologies and implementing new features became increasingly challenging and, in some cases, unfeasible.

### How did we do it?

**Fig. 2.**
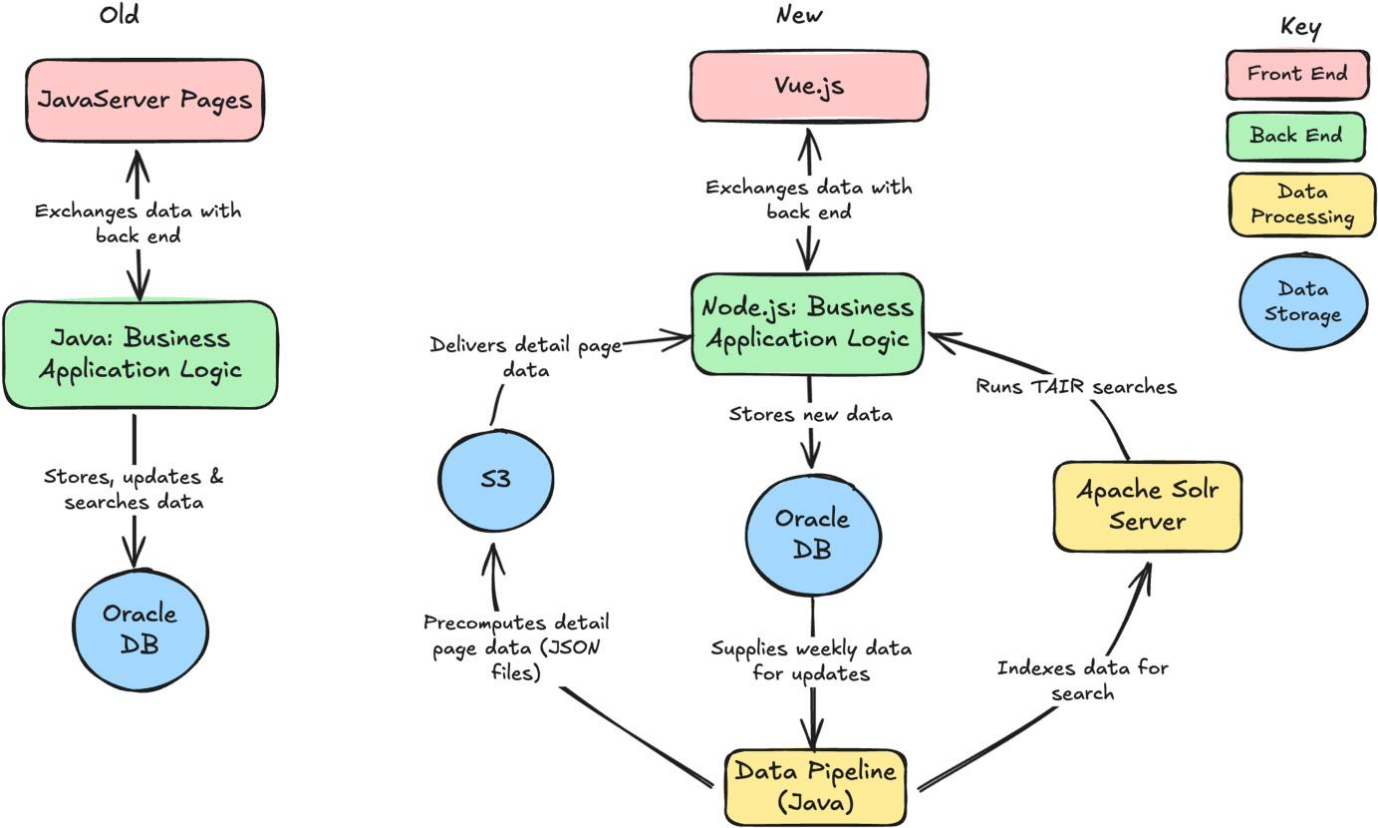
Old vs. New Architecture of the TAIR UI. The old codebase was a single, large codebase (monolithic) that was tightly integrated and had to be deployed as a single unit. The new architecture utilizes microservices and containerization for more flexibility and scalability.

We adopted a microservice-based architecture (Fig. 2) for our overhaul, enabling independent scaling and fault isolation of individual components. The new UI is based on a Single Page Applications (SPAs) framework. SPAs offer a dynamic and responsive user experience by enabling seamless interaction on a single web page, reducing the need for reloading and improving performance. To improve search, we migrated from a relational query layer to Apache Solr, which provides schema-flexible indexing and distributed retrieval. We have improved retrieval speed for complex queries by around 30% because of this upgrade.

To further reduce user latency on detail pages, we introduced a preprocessing stage that consolidates data from multiple related tables into precomputed, denormalized views. This skips complex query joins to our database for every page load. We run a data syncing step every week to keep the Detail Page data updated with the data that is added, deleted, or updated in our curation database every day.

All services are containerized with Docker. Containerization ensures a consistent environment across development and production, simplifies continuous delivery of updates, and allows new functionality to be added as isolated modules with minimal impact on existing services. This has sped up development time for our engineers to deploy fixes and new features at a much faster rate than before.

By transitioning to a modular, flexible architecture, we have streamlined operations and improved scalability. This new setup not only optimizes current features but also allows us to introduce new capabilities with ease. Shifting from costly monolithic servers to efficient, scalable servers reduces operational costs, a key attribute of being good fiscal stewards of user-backed funding and an important factor in ensuring TAIR's long-term viability.

The replatforming project had many moving parts. Search and browse needed to be updated, tools needed migration, and even registration was affected. Tasks were managed using the Atlassian tool JIRA, and product specifications were documented using another Atlassian tool, Confluence.

The development and testing process was deeply informed by user testing and iteration. After coming up with very detailed specifications for the locus page and developing the software for that module, a similar design was applied to all other detail pages. Testing proceeded in several stages, first internally with TAIR curators, looking not only at design but also at data integrity. When all features had been migrated to internal satisfaction, a beta version of the website was announced and made available to the public. An open comment period allowed for feedback collection and immediate response. We then pushed the beta version into production and replaced the old site. The entire development process forced us to test the functionality of the old website and then migrate only those parts that were still in use and made sense to move over. We identified and resolved legacy issues, implementing measures to prevent their recurrence in the new site.

Since the May 2024 release, we continue to improve the site, with direct user input gathered by email and in person at conferences. Information about updates, such as bug fixes and enhancements, can be found in the release notes (https://phoenixbioinformatics.atlassian.net/wiki/spaces/COM/pages/42217301/Release+Notes).

## Significant changes to the UI and functionality

### Changes to data type searches and detail page displays

#### Changes to the general search

The general search function appears in the header of the TAIR website and provides a simple search for different datatypes, including Genes, Clones, Keywords, Proteins, People, Ecotype, Germplasms, Polymorphisms, Markers, Vectors, and Transposons. Most searches are by name or accession, with the exception of Gene search, which also queries descriptive information. Some of the changes made include reordering results that exact matches are returned first and limiting the general search to specific data types in TAIR.

For example, a search for AG will return *AGAMOUS* as the first result but will also include *AGAMOUS-LIKE* genes. Future plans for the general search include enabling searching with UniProt IDs.

#### Changes to advanced searches

We took the opportunity to modify the search interfaces and, in some cases, the underlying functionality for some of the advanced searches. Where possible, we simplified the search interfaces, eliminating options that were no longer relevant.

We made changes to the DNA/Clone and Seed/Germplasm searches that had been delayed since TAIR stopped providing catalog/ordering functions for the *Arabidopsis* Biological Resource Center (ABRC) in May 2019. Previously, users could search for all ABRC stocks via TAIR, but now the ABRC maintains its own website and ordering functions (https://abrc.osu.edu/). The updated TAIR DNA/Clone search is now limited to finding clones and vector records in TAIR. Users wishing to find cDNA libraries, clones, and other DNA stocks are now directed to the stock centers themselves.

Users searching for Seed/Germplasm resources in TAIR should be aware that TAIR no longer synchronizes data with ABRC. While we still offer the “ABRC stock” filter and links out to stock centers, users should perform independent searches of the ABRC, Nottingham *Arabidopsis* Stock Center (NASC), or RIKEN catalogs to view the most current available seed stocks.

We modified the search by location in the Gene, Germplasm, Polymorphism/Allele, DNA/Clone, and Seed/Germplasm search interfaces to only search within the reference genome. Previous options included genetic maps and BAC assemblies, which were deemed no longer relevant. Another significant interface change was enabling bulk querying of Genes and Proteins as described in the section on Bulk Downloads below.

In some cases, we removed some search functions entirely. We eliminated the Microarray Experiment and Microarray Expression Searches because TAIR stopped accepting microarray data in 2006, and no new data has been integrated since then. Instead, researchers should submit array data to generalist repositories such as EBI's Array Express or NCBI GEO. In order to enable access to the historical data in TAIR, we created a browseable list of all the microarray experiments held in TAIR (https://www.arabidopsis.org/browse/microarray_experiments).

#### Changes to search results display

We modified the search results pages for each of the data types to improve usability. All results pages now have the option to sort on different columns. For example, one can perform a search for Polymorphisms/Alleles where the phenotype contains “embryo lethal” and then sort the results based on polymorphism type (e.g. substitution, T-DNA insertion). Previously, users had to select the number of results per page to display on the search page. Now this option is presented on the results display. So, if a search returns 75 results, users can choose the appropriate number to display (e.g. 100 results/page) to see the entire list. Users can check off specific items from the list or check all and download the table in tab-separated values (.tsv) format.

#### Locus and other detail page redesign

**Fig. 3.**
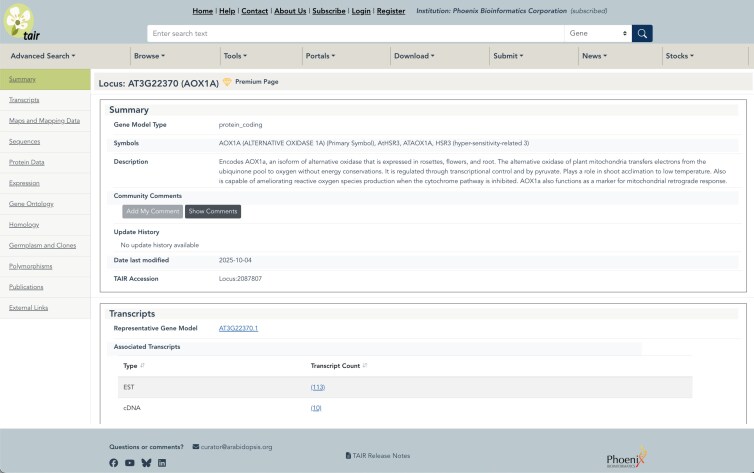
New locus page featuring left side navigation menu. Clicking on the topic area displays the relevant part of the page and reduces scrolling.

A main goal of our UI redesign was to improve the data detail pages. In particular, we wanted to update the TAIR locus page, which is our most viewed data type, with over 200K page views per month. The locus page collects all the information related to a given locus and presents a unified view and launching point for a deep dive. After several rounds of collecting user feedback, we implemented the following changes to improve navigation and visualization. First, we added a side navigation menu to make it easier for users to quickly know what types of information is available on the page and to jump directly to those areas (Fig. 3). Second, we reorganized the different data “bands” into main content areas including Summary, Transcripts, Maps and Mapping Data, Sequences, Protein Data, Expression, Gene Ontology, Homology, Germplasm and Clones, Polymorphisms, Publications, and External Links. Third, we added sorting to tabular data fields within the sections. This enables users to sort on fields such as publication date or polymorphism type directly from the locus page. This sorting function is also implemented in all search results pages. All the other data detail pages (e.g. Protein, Polymorphisms) went through a similar overhaul with the addition of the side navigation and reorganized sections aimed at improving access to each page's content.

#### Changes to the bulk query and data retrieval

**Fig. 4.**
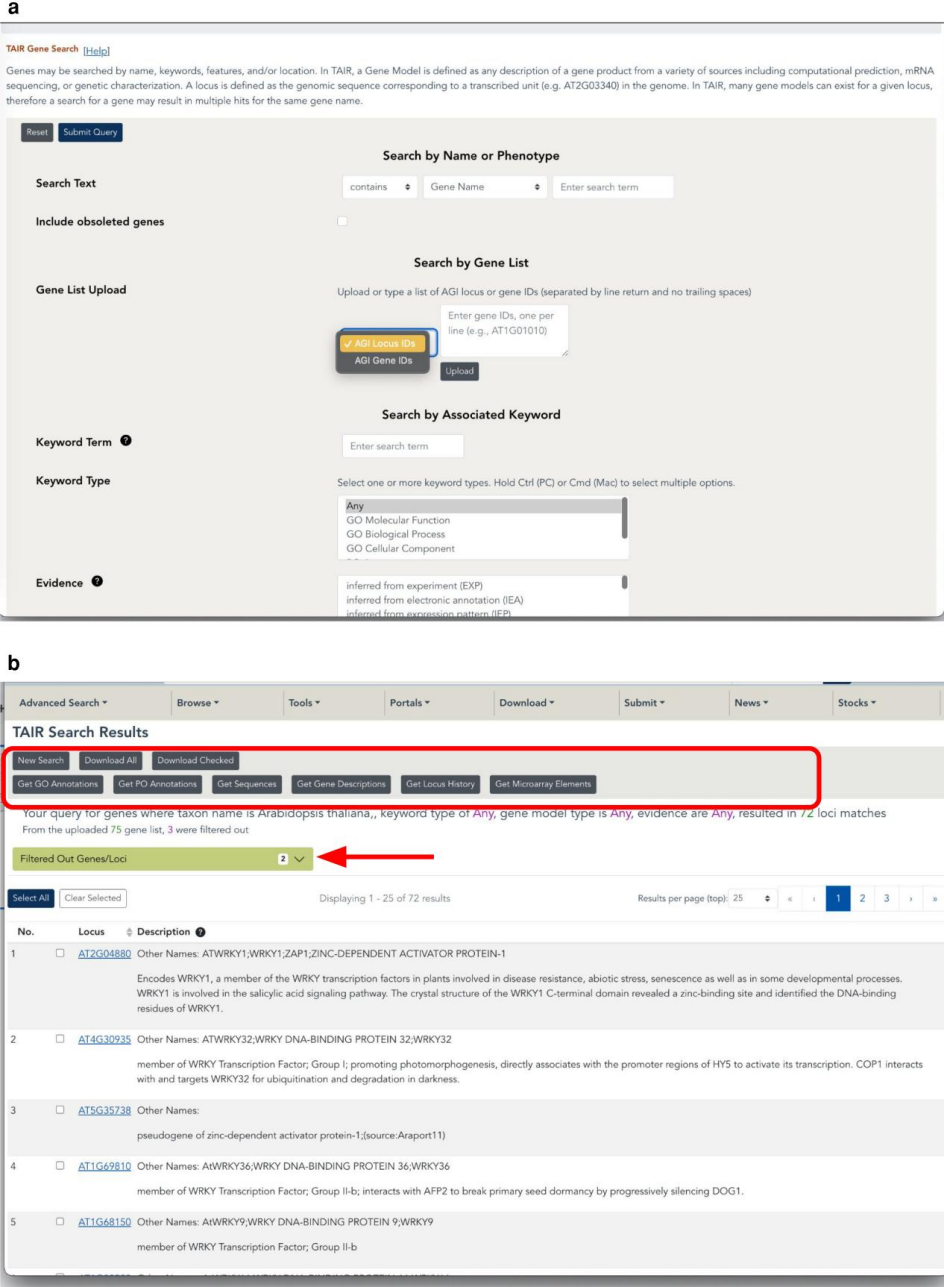
New bulk gene search and download. a) The interface for the advanced gene search, including the bulk AGI Locus/gene ID upload. Users can upload a list of locus IDs (e.g. AT3G23320) or gene identifiers (e.g. AT3G23320.2). b) Search results page showing the additional download options (boxed area). The results also include an output (arrow) showing any identifiers that were not included in the results (e.g. due to typos).

We used the overhaul project to make changes to some of the most popular tools at TAIR—the bulk data downloads. These tools took lists of AGI locus identifiers as input to obtain datasets such as gene descriptions, Gene Ontology/Plant Ontology annotations, or sequences. As part of the overhaul, the functions of these standalone tools were incorporated into the Advanced Gene and Protein search and retrieval system. Users can now upload a list of locus or gene IDs directly into the Advanced Gene or Protein search (Fig. 4). From the search results, one can select the entities and associated data one wishes to retrieve in bulk. Bulk download options from the Gene search include Get GO annotations, Get PO annotations, Get Sequences, Get Gene Descriptions, Get Locus History, and Get Microarray Elements.

The Advanced Protein Search also enables users to upload a list of locus or gene identifiers and then apply search parameters such as protein length and InterPro domains, among other features, and then download the list of results. It differs from the gene search in that there are no additional options for data retrieval from the results page other than the primary protein search results.

#### Legacy and other software package migration

TAIR hosts several analysis and visualization tools that also need to be migrated to the new platform. BLAST and PatMatch have been migrated; Motif Analysis, Functional Categorization, and Chromosome Map Tool will follow. Other legacy tools will be retired as their functionalities are replaced by more modern software. Maintaining legacy software is costly and, in some cases, not possible as the necessary knowledge and programming languages become obsolete. For example, GBrowse has been effectively replaced by JBrowse and JBrowse2. TAIR's in-house genome browser, SeqViewer, whose codebase has been static for over 15 yrs, will be retired, as we will not be able to update the software with the TAIR12 genome data. This was a difficult decision because SeqViewer has a dedicated fan base that finds some of the features of the nucleotide sequence view invaluable. Some of the unique features users like are viewing T-DNA insertion sites on the nucleotide sequence and being able to easily visualize and copy upstream/downstream intergenic sequence regions. We have been communicating with the JBrowse2 developer community to encourage and support the addition of these SeqViewer features into the JBrowse2 platform.

#### Help and other documentation migration to confluence

TAIR maintains many content pages that are not generated from the database, which need to be regularly updated and maintained. Rather than deploy a separate content management system to maintain these pages, we opted to use Confluence (Atlassian Software, already in use for internal knowledge management) to manage the documents for the TAIR website. Curators can quickly and directly update content in Confluence, which helps reduce maintenance costs. Pages in Confluence include the Help documents (https://www.arabidopsis.org/help), the About pages (https://www.arabidopsis.org/about/), and the Portal pages (https://www.arabidopsis.org/portals/overview). Most pages do not get updated that often with the exception of the Arabidopsis Community pages, where users can find updated lists of resources (e.g. stock centers, gene expression databases, and tools), TAIR release notes (https://phoenixbioinformatics.atlassian.net/wiki/spaces/COM/pages/42217301/Release+Notes), and TAIR12 reannotation (see next section) progress notes. While we have tried to ensure that all the pages have been properly updated and linked, there are undoubtedly links that have been broken. We fix those as we become aware of them.

#### Web accessibility compliance

Replatforming TAIR to more modern technology allowed us to comply with accessibility requirements from US state universities that arose from the United States Department of Justice (DOJ) ADA Title II ruling (https://www.ada.gov/resources/2024-03-08-web-rule/). The ruling was added to the U.S. Federal Register on April 24, 2024, and requires that public entities ensure their websites, mobile apps, and other digital properties are compliant with Web Content Accessibility Guidelines (WCAG) 2.1 Level AA by April 24, 2026. While the legacy TAIR would not have been required to comply with these standards, TAIR wanted to be proactive and better equipped to meet the evolving needs of the scientific community. Our new software makes compliance and adaptation to changing requirements possible.

## The reannotation of the *A. thaliana* Col-0 reference genome: TAIR12

The *A. thaliana* genome reannotation project, TAIR12, represents a significant milestone in the plant biology research landscape. This ambitious and community-driven initiative, led by the TAIR team at Phoenix Bioinformatics, advances the accuracy and depth of genome data for this vital model organism. *A. thaliana* was the first plant genome that was completely sequenced (Arabidopsis Genome Initiative 2000) and has served as a reference for other plants for over 25 yrs (Sun et al. 2022). The last update to the genome, called Araport11, was released almost 10 years ago (Cheng et al. 2017). Since then, sequencing, assembly, and genome annotation technology have improved dramatically, providing the opportunity to generate a high-fidelity, gapless genome sequence assembly and an extremely well-supported structural gene annotation anchored to it.

**Fig. 5.**
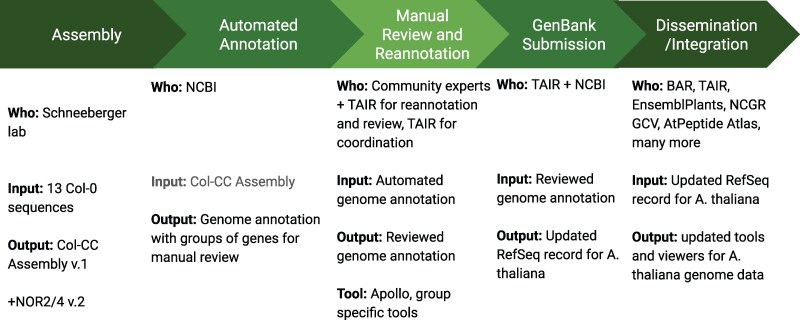
Overview of the *A. thaliana* TAIR12 genome reannotation project from genome assembly to dissemination.

This project (Fig. 5) consisted of five phases: Assembly, Automated Annotation, Manual Review and Reannotation, GenBank Submission, and Dissemination/Integration. A manuscript describing the first four phases in detail is in preparation by the TAIR12 team. This reannotation is unique because it had no specific grant funding to pay for the effort. Instead, it relied on a voluntary community curation effort with the infrastructure, organization, and bioinformatics support contributed by TAIR and Phoenix. Over 100 scientists from all over the world with expertise in many different areas gave their time and skill for the good of the larger research community. The Schneeberger laboratory at the Max Planck Institute in Cologne assembled the new chromosome backbone, the foundation of the reannotation, from 13 high-quality long-read genome sequences of the reference ecotype Col-0 (TAIR12 paper, in preparation). The Pikaard laboratory at Indiana University provided the sequences of the nucleolar organizer regions (NORs) at the tips of chromosomes 2 and 4 (Fultz et al. 2023) so that we could create a true complete genome of Col-0. The US National Center for Bioinformatics (NCBI) ran its Eukaryotic Genome Annotation Pipeline (https://www.ncbi.nlm.nih.gov/refseq/annotation_euk/process/) on this assembly and provided the resulting files to the TAIR team for review. The TAIR team spearheaded and coordinated the expert review of over 10% of the predicted protein-coding genes, focusing on those that had changed since the Araport11 annotation. Other collaborative groups took on the task of full annotation or reannotation of the transposable elements, lncRNAs, ribosomal RNAs, repeat elements, centromere, telomere, and NORs. The final integration and quality control of all newly annotated and reviewed genes and other sequence elements, and subsequent submission of the finished annotation to GenBank, was taken on by the TAIR team.

Taking on this project required developing new internal expertise. At the time we embarked on the reannotation, the TAIR team had no in-house experts or existing pipeline for structural annotation. Team members who had worked on the TAIR6 through TAIR10 releases were no longer with the organization, and we had to start from scratch. Fortunately, we were able to tap into the community of biocurators and bioinformaticians who had completed similar projects for other genomes using similar tools and were willing to share their expertise. There was also ample documentation for Apollo, both in text and video form, that we consulted as we set up the environment for manual review. Nonetheless, the initial month of the setup felt very much like learning to fly the plane while building it. Our particular genome and the specific datasets we were using for validation provided their own idiosyncrasies that needed to be addressed.

The community of manual reviewers came together in training sessions and office hours on Zoom to learn and then work out critical and common questions. The expert review process required meticulous attention to detail while examining the supporting data. It involved primary and secondary checks on updated genes, comparisons with external protein databases like Peptide Atlas, and the resolution of gene overlaps or sequence issues. Sixty-five individuals contributed to the review effort.

Table 2 shows the differences between the various annotation releases from the initial one in the year 2000 to TAIR12 today. The current TAIR12 annotation will continue to shape plant genomics by providing the most comprehensive and reliable reference genome that has ever been available. The reannotation serves the *Arabidopsis* research community and strengthens the foundation for comparative genomics and functional studies in other plant species.

**Table 2.** *Arabidopsis* genome annotations 2000 to 2025.

Version	Size of genome (chr. 1–5)	Protein-coding genes	Transposons and pseudogenes	Alternatively spliced genes
**TAIR12 (2025)**	145.2 Mb	26,868	19,364	5,701
**Araport11 (2016)**	119.1 Mb	27,655	4,853	10,695
**TAIR10 (2010)**	119.1 Mb	27,411	4,827	5,885
**TAIR9 (2009)**	119.1 Mb	27,379	4,827	4,626
**TAIR8 (2008)**	119.1 Mb^a^	27,235	4,759	4,330
**TAIR7 (2007)**	119.1 Mb	26,819	3,889	3,866
**TAIR6 (2005)**	119.1 Mb	26,541	3,818	3,159
**TIGR5 (2004)**	119.1 Mb	26,207	3,786	2,330
**TIGR4 (2003)**	119.1 Mb	27,170	2,218	1,267
**TIGR3 (2002b)**	119.1 Mb	27,117	1,967	162
**TIGR2 (2002a)**	119 Mb	26,156	1,305	28
**TIGR1 (2001)**	119 Mb	25,554	1,274	0
**Nature (2000)**	119 Mb	25,498	NA	NA

^a^Several updates were made to the genome sequence without changing the length of the chromosomes. NA = not annotated.

The assembly was made available through GenBank. The genome assembly including the NORs (Col-CC, Columbia-0 Community Consensus, GCA_028009825.2) was released on October 18, 2023. The genome annotation (TAIR12) was submitted for review through the GenBank portal on April 28, 2025, and is currently in the processing phase. Any resources, including TAIR, that want to incorporate the new annotation should download the information directly from GenBank, using that as the central source of truth. This ensures that the work that was done as a community effort is available without any restrictions.

The reannotation project was built based on community needs and realized through community support. Through the subscriptions of institutions and individuals all over the world, TAIR was able to provide the computing infrastructure, organizational infrastructure, and bioinformatics expertise to serve as the foundation. Through the contribution of subject matter expertise from scientists all over the world, the scientific analyses and rigorous review of new data sets were made possible. The first community meeting was held by Zoom in late October 2022. The call solidified the need for the reannotation and solicited the buy-in from the necessary groups to make it happen. The initial timeline that had the complete annotation delivered to GenBank by January 2024, for a total of 14 mos of work, was ambitious.

As a group, we learned many lessons and offer these pieces of advice for any community wanting to embark on a similar annotation project.

1. Make a realistic timeline and then double it. Things will take longer than you think. Budget at least twice the amount of time. This is essential, especially when volunteers are involved.
2. Start working, even if you don't know exactly what you are doing yet. We learned how to install Apollo, load the genome and all the tracks, and how to edit gene structures from online documentation, YouTube videos, and calls to colleagues. This still didn't prepare us enough for the quirks of our particular genome.
3. Documentation and communication are critical. We hosted a set of webpages built in Confluence where we could post updates, training materials, and guides. We also created a shared Slack channel for immediate feedback and shared, searchable conversations. During the manual curation phase, we hosted regular Zoom office hours for live troubleshooting and discussion. Having multiple ways of keeping the distributed team in touch and aware of changes kept the project moving forward.
4. Manage expectations. Set goals, but do not get discouraged when they are not met. Some things are out of one's control.
5. Ask for help. It was incredible how many people were willing to contribute. The criticality of the end product was powerfully motivating.

## User-backed funding, 12 yrs later

Model organism and other databases face significant challenges in attracting and retaining long-term funding, which ultimately impacts reproducibility, data accessibility, and preservation (Bellen et al. 2021; Global Biodata Coalition 2025). In 2013, following the termination of funding from the National Science Foundation for TAIR, Phoenix Bioinformatics launched an experiment to see if user-backed funding was a viable alternative to episodic and unreliable grant funding (Reiser et al. 2016). In 2024, TAIR celebrated its 25th year of existence and 10 yrs of user-backed funding; milestones made possible through the support from the global research community. The user-backed funding model distributes the cost among users, with the most active users contributing the most. As of August 2025, the types of subscribers include national (1), academic/nonprofit institutional (220), consortia (10), individual (517), and corporations (5).

**Fig. 6.**
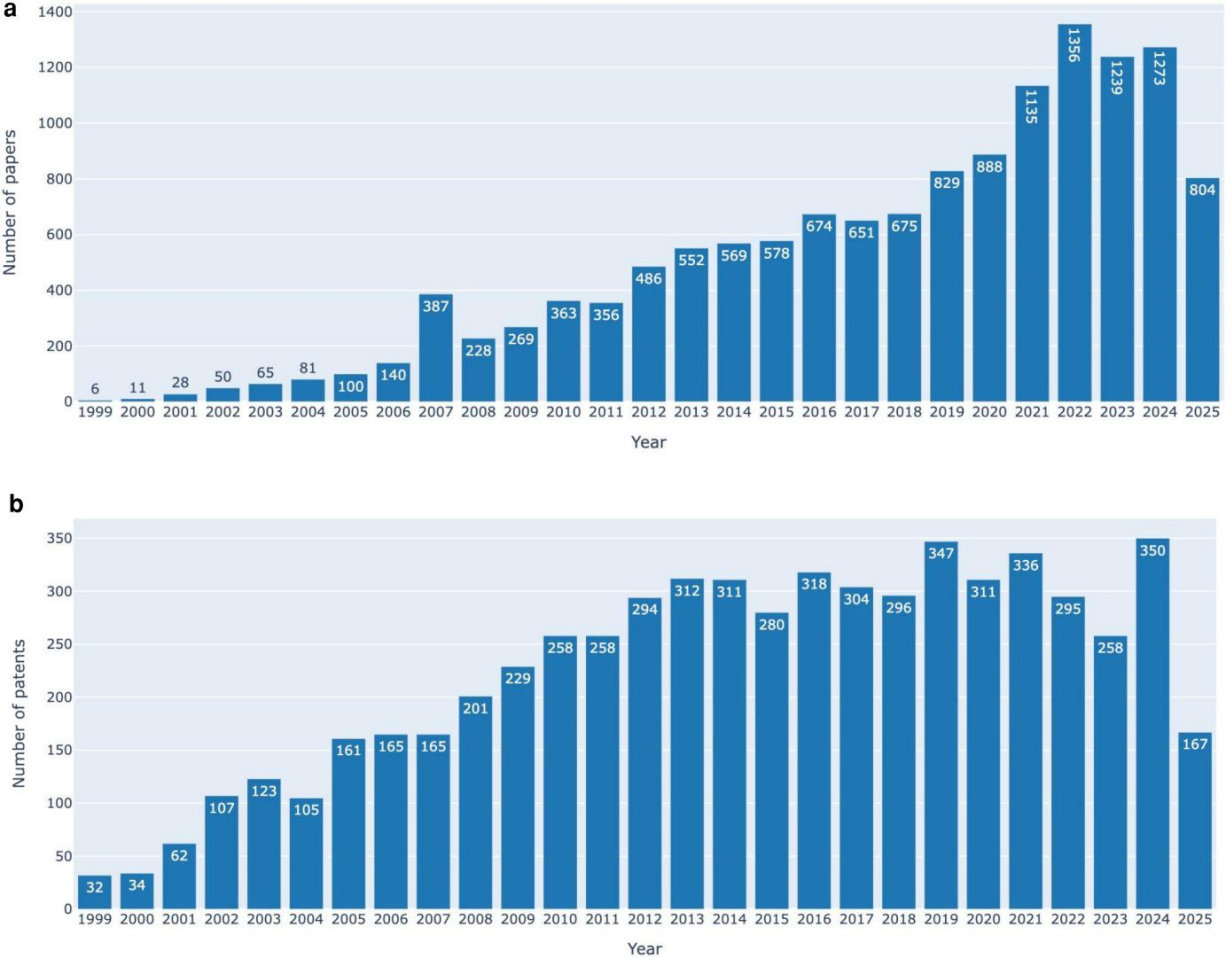
a) The number of TAIR citations over time. A plot of the number of times “TAIR”, “www. arabidopsis.org”, or “The Arabidopsis Information Resource” was found in the full text of papers in European PubMed from 1999—August 2025. b) The number of times “TAIR” or “www.arabidopsis.org” was mentioned in patents in SureChEMBL over time. Data and plot generated using the Molecular Biology Database Metrics CoLab (Insana et al. 2023).

Since launching the nonprofit user-backed funding model, TAIR has been sustainable with revenues sufficient to continue curation (albeit at a slower rate than at peak NSF funding), maintain and upgrade software systems, and support initiatives such as the TAIR12 genome reannotation and platform upgrades described in this paper. The return on investment includes weekly updates to the database and quarterly releases of current (subscriber) and year-old (public) datasets. So far, the experiment has validated the continued need for the TAIR resource and the TAIR-specific user funding model. Evidence that TAIR continues to be a valuable resource for reusable research data is supported by the usage (well over >200,000 locus page views each month in 2024 to 2025, increasing number of mentions in journal articles (Fig. 6a) and patents (Fig. 6b). For TAIR, the user funding model has provided a distributed and reasonably stable source of funding for the past 12 yrs. While less dependent on grant funding, long-term sustainability remains dependent on a healthy, well-funded research ecosystem.

### Changes to the individual subscription model

Based on our experience over the past 12 yrs, we have made some tweaks to the subscription model. When TAIR began subscriptions, we determined that the most equitable way to charge academic and nonprofit institutions was based on usage rather than size, as many other digital resources do. To be consistent with the institutional usage-based funding model, TAIR individual access was updated in 2025 to usage based on “units.” Individuals who are registered and linked to their ORCID profile get an allowance of 50 complimentary “units” that resets annually. Detail page views (e.g. Locus, Polymorphism, Germplasm) are a single unit, while files downloaded from the Subscriber_Data_Releases are 25 units each. Should the annual complimentary allowance not be sufficient, individuals can purchase additional “buckets of units” that are good for a year. All subscribed users still have access to the most current data and tools on the website, quarterly releases, and custom datasets. For those who do not subscribe, there is a complimentary annual usage allowance and the public datasets of 1-yr-old data.

When TAIR transitioned to subscription support, one of the ways we tried to mitigate access restrictions was by providing complimentary teaching access for instructors who were at institutions that could not or would not subscribe to TAIR (Reiser et al. 2016). Since 2014, complimentary access has been granted to instructors at over 150 institutions (mostly colleges and universities, but some high schools and middle schools as well) in over 40 countries. With the new individual model, teachers and their students have the same access to the 50 complimentary units as all other TAIR users. If the 50 complimentary units are consumed during the course period and more are needed, requests for additional teaching credits should be sent to subscriptions@phxbio.org, and they will be readily granted.

## AI and the future, challenges, and opportunities

### The role of TAIR and other knowledgebases in data democratization

Curated knowledgebases such as TAIR are essential for achieving the aim of ensuring data is FAIR and ready for developing AI capabilities (Poveda et al. 2025). Curated knowledgebases and repositories ensure that data is well described with accurate metadata, properly formatted, machine-readable, standardized, and easily discoverable. These high-quality curated data have been used to successfully train AI models such as AlphaFold, which relied heavily on structured, curated data from the Protein Data Bank rather than information gleaned from unstructured data (Dessimoz and Thomas 2024). Researchers who want to or have used TAIR data for developing AI models/tools should contact us by email at curator@arabidopsis.org to access AI-ready datasets. Data scraping by AI bots is discouraged as this tends to lead to servers being overwhelmed and services being taken down (see section below). TAIR's public data is released under a CC-BY license, and researchers should follow the instructions for citing TAIR provided in the associated README files. Citing databases in publications is important as it helps us to quantify the impacts of our resources and then communicate that impact to all our stakeholders.

### How researchers can help make their data more FAIR

Unfortunately, it is simply not possible for database curators to keep up with the sheer volume of data, and researchers must take a more active role in curating their own data. Most researchers, at least in the USA, are well aware of new requirements for data sharing for federally funded research as outlined in the “Nelson Memo” in 2022 (https://bidenwhitehouse.archives.gov/wp-content/uploads/2022/08/08-2022-OSTP-Public-Access-Memo.pdf). However, “open data” is not necessarily FAIR. Authors, editors, and reviewers can enable data curation, thus improving the FAIRness of published research (Reiser et al. 2018). TAIR provides some guidelines specific to the *Arabidopsis* community (https://phoenixbioinformatics.atlassian.net/wiki/spaces/COM/pages/42213363/Tips+for+making+your+published+data+about+Arabidopsis+genes+more+FAIR+Findable+Accessible+Interoperable+and+Reusable). Existing and future AI tools can and will accelerate curation, and large language models are being deployed to tackle gene function prediction. Perhaps the single most important thing researchers can do to assist is to include unique locus identifiers (the AGI locus identifiers) in their publications. AGI locus IDs are identifiers for a specific locus (e.g. AT1G13750) and, when coupled with the genome annotation version (e.g. AT1G13750_TAIR12), can be used to unambiguously identify a gene, whereas gene symbols (e.g. PAP1) are frequently reused both within and across species. The consistent use of standard, unambiguous, persistent identifiers ensures that data can be properly associated by humans and machines.

### Use of AI in biocuration

Keeping pace with the literature curation has always been a challenge that has been exacerbated by the inverse relationship between funding for curation and the number of papers published annually. Biocuration at TAIR and other model organism databases (MODs) has been facilitated by using a software to assist at various stages of the literature curation workflow. For many years, TAIR's literature curation pipeline has used in-house software such as PubSearch (Yoo et al. 2006) and Paper Grab. PubSearch incorporates simple string matching for entity recognition. We have also used the Textpresso (Van Auken et al. 2012) NLP tool for making cellular component Gene Ontology associations. Other MODs have produced newer tools to facilitate curation that rely upon natural language processing and purpose-built LLMs. These tools are not intended to supersede but rather to assist the biocurator by automating as much as possible of the triage and fact extraction. With these tools, curators can more easily validate bioentities and make annotations, thereby increasing curation throughput. Some of the tools being developed by MODs under the Alliance for Genome Resources are likely candidates for adoption by TAIR. These include an AI-assisted literature curation platform for authors (Arnaboldi et al. 2020) and carefully designed and vetted tools for constructing gene summaries based upon annotations stored in the database (Arnaboldi et al. 2020; Kishore et al. 2020) and linked literature (Kishore et al. 2024). We would carefully assess the quality of any tools and data to ensure that annotations are of high quality. Any AI-generated summaries or annotations would be clearly distinguished as computationally generated. We take our responsibility as stewards of a trusted community resource very seriously and are committed to ensuring the high quality of data we provide.

### Battling scraping AI bots

The practice of scraping database websites by AI bots can result in servers being overwhelmed, leading to downtimes that limit access by researchers (Kwon 2025). We observed increasing volumes of nonhuman traffic and scraping bots from AI companies, which degraded performance for real users and did not comply with our data-use policy. To solve this, we implemented an application-layer defense using AWS Web Application Firewall Bot Control. Suspected clients receive challenges–response tests like Captcha to prove they are human, and we use regularly updated blacklists to block certain traffic which are known to be associated with scraping bots. This setup significantly reduced the bot traffic directly hitting our website, as it was blocked at the network edge itself, keeping our data secure and performance intact.

### AI integration at TAIR—a peek into the future

With the replatforming behind us, we are now beginning work on incorporating AI into TAIR, starting with a chatbot. The initial use cases were gathered at conferences, by email, and through a 2024 user survey. Our initial goal is to enable users to navigate more quickly through the website to reach their desired data, whether that be gene-related or not. In time, we want to enable more complex queries such as “find all substitutions that are associated with a mutant phenotype.” AI integration at TAIR could facilitate experiment design, literature summarization, and phenotype exploration—integrating vetted data and verified publications on genes, mutants, and interactions. Additional possibilities include a facilitated search of TAIR help documentation for beginning users and connecting to other resources like the ABRC using application programming interfaces or perhaps model context protocols, standardized protocols through which generative AI (GenAI) systems can use tool calling to interface with external information and applications (Hou et al. 2025; Kuehl et al. 2025; Anthropic 2024) for a consolidated search that is broader than only TAIR. Our initial experiments in integrating AI have focused on evaluating different out-of-the-box solutions and comparing them with bespoke combinations of LLMs, document storage, and hosting solutions. We will most likely be using an AI agent framework as we proceed, although the technology and options are evolving so quickly that choices described in this paper may be out of date by the time of publication.

## Data Availability

The website URL is www.arabidopsis.org. Cumulative data files with information on gene function, publication links, germplasm, and phenotype information are released every quarter (beginning of January, April, July, and October). Subscriber Data Releases contain data updated within the last 12 mos and are available at this URL to those with current subscriptions to TAIR:https://www.arabidopsis.org/download/list?dir=Subscriber_Data_Releases. The use of these files is governed by the Terms of Use, full text available here: https://phoenixbioinformatics.atlassian.net/wiki/spaces/COM/pages/42216430/TAIR+Terms+of+Use. After a year, the Subscriber Data Releases are moved into the Public Data Releases folders at this URL: https://www.arabidopsis.org/download/list?dir=Public_Data_Releases. All files in the Public_Data_Releases folder are made available to the public under the CC-BY 4.0 license (https://creativecommons.org/licenses/by/4.0/).
